# The impact of workplace risk factors on the occurrence of neck and upper limb pain: a general population study

**DOI:** 10.1186/1471-2458-6-234

**Published:** 2006-09-19

**Authors:** Julius Sim, Rosie J Lacey, Martyn Lewis

**Affiliations:** 1 Primary Care Musculoskeletal Research Centre, Keele University, Staffordshire ST5 5BG, UK

## Abstract

**Background:**

Work-related neck and upper limb pain has mainly been studied in specific occupational groups, and little is known about its impact in the general population. The objectives of this study were to estimate the prevalence and population impact of work-related neck and upper limb pain.

**Methods:**

A cross-sectional survey was conducted of 10 000 adults in North Staffordshire, UK, in which there is a common local manual industry. The primary outcome measure was presence or absence of neck and upper limb pain. Participants were asked to give details of up to five recent jobs, and to report exposure to six work activities involving the neck or upper limbs. Psychosocial measures included job control, demand and support. Odds ratios (ORs) and population attributable fractions were calculated for these risk factors.

**Results:**

The age-standardized one-month period prevalence of neck and upper limb pain was 44%. There were significant independent associations between neck and upper limb pain and: repeated lifting of heavy objects (OR = 1.4); prolonged bending of neck (OR = 2.0); working with arms at/above shoulder height (OR = 1.3); little job control (OR = 1.6); and little supervisor support (OR = 1.3). The population attributable fractions were 0.24 (24%) for exposure to work activities and 0.12 (12%) for exposure to psychosocial factors.

**Conclusion:**

Neck and upper limb pain is associated with both physical and psychosocial factors in the work environment. Inferences of cause-and-effect from cross-sectional studies must be made with caution; nonetheless, our findings suggest that modification of the work environment might prevent up to one in three of cases of neck and upper limb pain in the general population, depending on current exposures to occupational risk.

## Background

Musculoskeletal pain in the neck and upper limbs is common; population studies suggest that 6–48% of adults have pain in one of these areas [1-4]. Identifying and acting on modifiable or preventable risk factors for such common painful conditions would significantly improve the health of adult populations. Musculoskeletal disorders are one of the most frequent reasons for long-term sickness absence [5], and those of the neck and upper limb account for approximately three-quarters of work-related musculoskeletal disease seen by UK rheumatologists [6]. Occupational factors therefore provide a potentially important target for population prevention strategies.

Most studies suggesting an association between neck and upper limb pain and workplace factors have occurred in specific occupational settings [7-10]. However, such studies do not estimate the overall burden of work-related neck and upper limb pain within a general population, or compare this burden between different types of occupational exposure. Furthermore, a workplace sampling frame may underestimate the population risk associated with occupational exposure through the "healthy worker effect". The few published general population studies have mostly investigated occupational associations with pain in single neck or upper limb areas [11-13], rather than in this region as a whole. One recent population study [14] assessed the association of repetitive strain injuries and workplace factors but drew on respondents' own causal attribution of injury to repetitive activities; associations were therefore mediated, and possibly confounded, by individuals' own assessment of causal factors. Estimates of the burden of work-related neck and upper limb pain require a sampling frame that captures a complete working age range, and includes both current and retired workers. The general population of North Staffordshire (over 342 000 adults) comprises a broad socio-economic background, and is a relatively static population in an area historically dominated by the pottery (ceramic) industry. Specific manual tasks within this industry involve prolonged postures and repetitive movements of the neck and upper limbs. This population therefore provides a 'natural laboratory' in which to examine the association of neck and upper limb pain with these tasks. We also investigated the association of neck and upper limb pain with psychosocial factors of the work environment, as previous studies have suggested associations with shoulder pain [11] and forearm pain [13]. If independent risk factors for neck and upper limb pain could be identified, and were modifiable or preventable, we could then estimate both the overall population impact of neck and upper limb pain and any differential impact between a local manual industry and other occupations within the population. From a public health perspective, this information may contribute to reducing the population burden of neck and upper limb pain.

The aims of this study were to (i) estimate the prevalence of neck and upper limb pain in an adult population with a substantial local manual industry, (ii) investigate associations between specific physical and psychosocial factors and neck and upper limb pain, and determine whether such factors explain any association between pottery work and neck and upper limb pain, (iii) estimate the proportion of cases of neck and upper limb pain in this general population attributable to such factors, and thus potentially amenable to primary or secondary prevention.

## Methods

### Subjects and design

Data were collected cross-sectionally by postal questionnaire in 2001–2002, using previously validated measures of neck and upper limb pain and occupational exposure [15]. North Staffordshire Local Research Ethics Committee approved the study, and recipients of the questionnaire consented to participation by completing the questionnaire and returning it to the principal investigator.

The study population was adults aged 18–75 years on the general practitioner (GP) database of the North Staffordshire District Health Authority, UK. We randomly sampled 10 000 adults equally from four age groups: 18–44, 45–54, 55–64, 65–75. Approximately 98% of the UK population are registered with a GP [16] and this sampling frame is considered representative of the general population [17]. Assuming an exposed:unexposed ratio of 1:5, this sample size would provide ≥ 96% power to detect a 5% difference in prevalence between exposed and unexposed, at a two-tailed 5% significance level.

We sent a reminder to all non-responders two weeks after the first questionnaire mailing, and sent remaining non-responders another questionnaire after a further two weeks.

### Questionnaire

#### Measurement of outcome

The focus of this study was non-specific neck and upper limb pain; we did not attempt to identify specific diagnoses in specific body areas. For the primary outcome, we used a pre-shaded manikin question, in which the neck, shoulder, arm and hand areas are treated as one region [18]. This question asked: "In the past 4 weeks, have you had pain that has lasted for one day or longer in any part of the shaded area?" (yes/no). Although interest in this study centred on the neck and upper limb region as a whole, we also asked respondents to shade a blank body manikin, from which we calculated the prevalence of pain in each of five areas within this region: neck; shoulder(s); elbow(s); forearm(s); hand(s) [19].

#### Measurement of exposure

We asked respondents to enter, in a grid, details of up to five most recent jobs (job title, area of work, start date, end date) held for at least 12 months. There was no limit to the length of time retrospectively in relation to which respondents could complete the grid. Occupational data were coded according to Standard Occupational Classification 2000 [20].

We asked whether or not ('yes/no' response) these jobs involved any of six activities – involving repetitive movements or sustained postures of the neck or upper limbs – on most or all days of the working week (which we deemed to represent substantial exposure):

• repeated lifting/carrying of heavy objects

• prolonged gripping/holding of an object

• bending the neck forwards for prolonged periods

• carrying out repeated movements with the fingers

• carrying out repeated movements with the wrists

• working with one/both arms at shoulder height or above.

From the jobs recorded, that held for the longest time was coded as the respondent's 'main job'. Psychosocial factors, relating to the main job only, were assessed by the following questions, on a five-point adverbial scale ('none of the time' to 'all of the time'):

• Can/could you control the way you worked in this job?

• Is/was your work physically demanding in this job?

• Do/did the tasks and activities that you perform/performed in this job change during your time in the job?

• Do/did you get job satisfaction from your work in this job?

• On the whole, are/were your supervisors/managers supportive?

Similar methods of collecting information on work activities and psychosocial factors – based on the control-demand model [21] – have been used previously [13,22,23].

#### Other questions

Other neck and upper limb symptoms assessed included severity of neck and upper limb pain (0–10 numerical rating scale, categorized: 0–5 mild; 6–7 moderate; 8–10 severe [24]), time since onset of neck and upper limb pain (five-point ordinal scale: less than 4 weeks; 1 to 6 months; more than 6 months but less than 12 months; 1 to 5 years; more than 5 years). The questionnaire also included questions about previous neck or upper limb injury, spare-time activities involving repeated movements of arms or hands, and demographics. We used the Townsend Deprivation Index as a measure of deprivation [25].

#### Reliability

The reliability of the pre-shaded manikin and of the measure of occupational history has previously been shown to be good [15].

### Statistical analysis

For the primary outcome, we analyzed the association between a one-month prevalence of neck and upper limb pain and pottery work, compared to non-pottery work, as main job. Cross-sectional associations with neck and upper limb pain were estimated by odds ratios (ORs) with 95% confidence intervals (CIs). Associations between potential risk factors and neck and upper limb pain were investigated through chi-square tests. Multivariable analysis used binary logistic regression. Two such analyses were performed: (i) partial regression – covariates were age, sex, Townsend category (ii) full regression – covariates were age, sex, Townsend category, pottery work as main job, all work activities and psychosocial factors.

To investigate selection bias, associations between neck and upper limb pain and work in the pottery industry were compared across the three waves of questionnaire response, to simulate the effects of non-response. This assumed that factors leading to non-response resemble those leading to late-response, and that late responders are therefore most representative of non-responders; a similar strategy has been used previously [26]. Confounding was investigated by adding to the full regression model: duration of main job, time since end date of main job, spare-time activities involving repeated movements of arms or hands, previous neck, shoulder, arm or hand injury, and comorbid pain marked on the blank body manikin. To account for recall bias, we repeated the full logistic regression (as described above) but additionally included interaction terms for each of the work activities and psychosocial factors with time since end date of the main job, quantified on a discrete numerical scale ranging from 0 years (current job is the main job) to 57 years.

The attributable fraction (AF) is the proportion of cases of a disease attributable to exposure to a particular risk factor or group of risk factors, or alternatively, the proportion of cases that would be prevented following elimination of these risks [27]. The *exposed AF *(AF_e_) is the fraction of those cases with pain who were exposed who would not have been cases in the absence of the exposure [28]. The *population AF *(AF_p_) is the fraction of all cases with pain (i.e. both exposed and unexposed) that would not have occurred in the absence of the exposure. The AF_p _takes into account not only the association between risk factors and outcome, but also the prevalence of the risk factors in the population as a whole, and therefore is a measure of the potential public health impact if the risk factors were removed [29]. The AFs were used to estimate the proportion of neck and upper limb pain cases attributable to exposure to identified significant risk factors. They were calculated from relative risks, derived from adjusted odds ratios using an appropriate conversion method [30], after establishing significant association in the earlier analysis. Estimates of the AF_p _and AF_e _for separate risk factors, and of the AF_p _for categories of risk factors (physical and psychosocial), were calculated by direct age-standardization to the North Staffordshire population [27,31].

Statistical significance was set at *p* ≤ 0.05 (two-tailed). Statistical analysis was carried out using SPSS version 12 (SPSS, Chicago, IL).

## Results

### Study population

A total of 5133 people replied to the questionnaire (2636 on initial mailing; 1191 after two weeks; 1306 after a further two weeks), a response of 53.5% adjusting for deaths and departures. A main job was identified for 4040 respondents (448 as pottery work; 3592 non-pottery work). The median (interquartile range) number of years worked in the main job was 18 (10–29); 20 (11–34) among respondents whose main job was pottery work, and 18 (10–28) among respondents whose main job was not pottery work. The median (interquartile range) number of years since the end date of the main job was 5 (0–12); 8.5 (3–20) among respondents whose main job was pottery work, and 4 (0–12) among respondents whose main job was not pottery work. Those reporting a main job in pottery work were older, more likely to be female, and more likely to be classed in deprived Townsend categories (*p *≤ 0.001 in each case).

### Neck and upper limb pain

A one-month period prevalence of neck and upper limb pain was reported by 50.5% (2539/5032) respondents (age-standardized prevalence 44.0%). Among the 2505 respondents who completed the question on first onset of neck and upper limb pain, responses were as follows: less than 4 weeks, 8.4% (*n *= 210); 1 to 6 months, 12.1% (*n *= 302); 6 to 12 months, 9.2% (*n *= 230); 1 to 5 years, 33.5% (*n *= 839); more than 5 years, 36.9% (*n *= 924). The prevalence of pain in each of the individual areas was as follows: neck, 24.0% (*n *= 1209/5032); shoulder(s), 31.7% (*n *= 1594/5032); elbow(s), 13.9% (*n *= 697/5032); forearm(s), 14.9% (*n *= 748/5032); hand(s), 19.2% (*n *= 966/5032).

Among the 3983 respondents with complete information for main job and the primary outcome, those with pottery work as their main job were more likely to report neck and upper limb pain in the previous month (258/438; 58.9%), than those with non-pottery work as their main job (1746/3545; 49.3%); a difference in prevalence of 9.6% (OR = 1.5; 95% CI 1.2, 1.8).

### Work activities and psychosocial factors

Neck and upper limb pain was significantly associated (*p *< 0.001 in each case) with all of the physical work activities (Table 1). There were significant associations between neck and upper limb pain and the following psychosocial factors: little job control (χ^2^_trend _= 94.8; 1df; *p *< 0.001); physically demanding work (χ^2^_trend _= 71.4; 1df; *p *< 0.001); little supervisor support (χ^2^_trend _= 33.7; 1df; *p *< 0.001). There was no association with change in tasks (χ^2 ^= 5.21; 4df; *p *= 0.266) or little job satisfaction (χ^2 ^_trend _= 1.7; 1df; *p *= 0.195) (Table 2).

### Multivariable analysis

In the partial regression model (adjusting for age, sex, Townsend category), prevalence of neck and upper limb pain was highest in the 55–64 age group, in females, and in the deprived Townsend category. All work activities and all psychosocial factors except change in tasks were associated with an increased prevalence of neck and upper limb pain, as was pottery work as main job (Table 3).

In the full model (adjusting additionally for pottery work as main job, work activities, psychosocial factors), three work activities were independently associated with higher prevalence of neck and upper limb pain: repeated lifting of heavy objects, prolonged bending of neck, working with arms at/above shoulder height. Two psychosocial factors – little job control and little supervisor support – were independently associated with increased neck and upper limb pain. Pottery work as the main job was not independently associated with neck and upper limb pain (Table 3).

To determine whether associations were consistent across the neck and upper limb region, the full multivariable model was repeated in each of the five individual areas. Table 4 shows the ORs for the work activities and psychosocial factors. Associations across the individual areas were generally consistent for the psychosocial factors. As regards work activities, the associations show some trends. For example, the ORs for prolonged bending of the neck decrease markedly from the most proximal to the most distal area (i.e. from neck to hand). Conversely, for prolonged gripping of object and repeated lifting of heavy objects, there were less marked decreases from distal to proximal (i.e. from hand to neck). The OR for repeated wrist movements was noticeably higher for the forearm area than elsewhere in the region. There were no marked trends for either 'repeated finger movements' or 'arms at/above shoulder height'.

### Potential bias

#### Selection bias

Response rates were higher among older people and women. Associations between work in the pottery industry and neck and upper limb pain across the three waves of mailing response were OR = 1.6, OR = 1.5 and OR = 1.4 respectively. Using logistic regression, there was no significant interaction (*p *= 0.754, adjusted for age, sex and Townsend category).

#### Confounding

After further adjusting the full model (in Table 3) for potential confounding factors (duration of main job; time since end date of main job; spare-time activities involving repeated movements of arms or hands; previous neck, shoulder, arm or hand injury; co-morbid low back, hip and knee pain), work activities and psychosocial factors were still significantly associated with neck and upper limb pain (repeated lifting of heavy objects (OR = 1.4); prolonged bending of neck (OR = 1.9); arms at/above shoulder height (OR = 1.2); job control (OR = 1.4); and supervisor support (OR = 1.3)).

#### Recall bias

Prevalence of neck and upper limb pain was higher in respondents whose main job was a past job (53.5%), compared to their current job (44.0%; OR = 1.3, *p *< 0.001 adjusted for age, sex and Townsend category). In the full multivariable model, there were no statistically significant interaction effects between the significant independent risk factors and time since end date of the main job.

### Attributable fractions

Table 5 shows age-standardized AFs for each of the three significant independent physical factors (repeated lifting of heavy objects; prolonged bending of neck; arms at/above shoulder height) and for the two significant independent psychosocial factors ('little job control' and 'little supervisor support', as defined in Table 3). These exposure-specific estimates are controlled for other risk factors in the statistical model. Estimates of both AF_e _and AF_p _are provided for presence versus absence of neck and upper limb pain.

In addition, estimates are given for 'severe' versus 'non-severe' neck and upper limb pain (categorized from the numerical rating scale for pain intensity), where 'non-severe' denotes 'no pain', 'mild pain' or 'moderate pain'. The significant independent predictors for this analysis comprised prolonged bending of neck, arms at/above shoulder height, job control and physically demanding work. We did not calculate AF values for risk factors in pottery versus non-pottery work as it was not an independent risk factor for neck and upper limb pain.

We also calculated AF_p _values for more than one risk factor, considered simultaneously. The age-standardized AF_p _for exposure to at least one of the three significant physical risk factors for neck and upper limb pain was 24%, and that based on reporting of at least one of the two significant psychosocial factors was 12%.

## Discussion

We have shown that respondents from a population sample who reported working in a common manual industry (pottery) were more likely to report neck and upper limb pain than non-pottery workers. However, pottery work per se was not independently associated with neck and upper limb pain, suggesting that our findings are not specific to a particular industry, and can be extrapolated to similar types of occupational exposure.

Within the general population studied, we estimated that 36% of cases of neck and upper limb pain were attributable to the significant risk factors identified in this study. Moreover, 43% of 'severe' cases of neck and upper limb pain were attributable to this same group of factors.

When associations with the risk factors were calculated for individual areas of pain, there were no discernible patterns in relation to the psychosocial factors. For the work activities, however, the magnitude of the ORs in the individual areas tended to reflect the nature of the work activity concerned (Table 4); for example, the strongest association for prolonged bending of the neck was with neck pain, and that for prolonged gripping was with hand pain. The trends observed for the work activities, and the lack of such trends for the psychosocial factors, are largely what one would expect, and reinforce the plausibility of the findings for the overall neck and upper limb region.

As regards individual risk factors, some substantial AFs were found. Approximately 14% of all cases (and 12% of 'severe' cases) of neck and upper limb pain in the population were attributable to prolonged bending of the neck. Although a work environment perceived to be physically demanding was not a significant independent risk factor for neck and upper limb pain, some 17% of 'severe' cases of neck and upper limb pain were attributable to this factor (Table 5).

The AF_p _figures in Table 5 are lower than the AF_e _figures because the former represent the proportion of all people in the total population of responders with neck and upper limb pain whose pain might be prevented by removal of the exposure. The AFs were generally higher for severe pain. This may reflect a greater likelihood of a specific causal mechanism for severe pain. However, we cannot exclude the possibility that respondents with severe symptoms are more likely to report possible exposures than those with less severe symptoms.

These occupational stressors therefore account for a sizeable population burden of neck and upper limb pain. Furthermore, these factors are in principle modifiable. Accordingly, from a public health perspective, there may be appreciable scope for preventive modification of the physical and psychosocial work environment to reduce the impact of neck and upper limb pain.

Although our study population was restricted to a particular UK region, our estimate of a one-month period prevalence of neck and upper limb pain of 44% is comparable to estimates from other studies [3,4]. Previous population studies of shoulder [11] and forearm [13] pain have found associations with working with hands above shoulder level [11] and lack of support [13]. A study of work-related repetitive injury – as defined by respondents and not related to a specified body region – found that high levels of psychological demand (hectic work and conflicting demands) and physical exertion in the workplace were significant predictors of work-related repetitive strain injury [14]. Similar psychosocial factors have also been shown to be associated with low back pain in an occupational setting [32]. This suggests that our estimates of population attributable risk are essentially generalizable, although the precise proportion of neck and upper limb pain in any general population sample which might be prevented by changes in the occupational environment will depend on the extent of current exposure to the occupational risks studied here.

In this study, there are potential biases. First, associations must be interpreted cautiously in cross-sectional studies. Hence, ORs were presented, as outcome may have pre-dated exposure, undermining the causal role of the exposure. However, when we restricted the analysis to respondents whose neck and upper limb pain did not predate a main job in pottery work, a significant association remained. This justifies the use of ORs to estimate the relative risks used to derive AFs for the physical and psychosocial risk factors. A similar approach to calculating AFs has been used previously [33].

Second, those who perceive themselves as being exposed to a potential risk factor may more readily report pain. We addressed this in the questionnaire, by gathering information on symptoms before that related to risk factors. Third, recall bias could inflate associations through respondents with pain erroneously identifying past risk factors in the main job. However, we are reassured on this, as there was a nonsignificant interaction between each of the significant independent risk factors and time since end date of main job, suggesting that the association between the risk factor concerned and pain did not differ according to how recent the main job was. Fourth, non-occupational factors may be alternative causes of neck and upper limb pain. However, the pattern of associations largely persisted after controlling for previous neck or upper limb injury and leisure activities involving repetitive arm or hand movements, reinforcing the role of occupational mechanisms. Fifth, the stability of the prevalence rates and of the associations across the waves of the questionnaire response makes it reasonable to assume that our estimates are representative. Finally, it must be acknowledged that exposure to risk factors was measured via self-report, rather than on the basis of an objective assessment. Moreover, respondents may have differed in their interpretation of adjectives such as 'repeated' and 'prolonged', such that these descriptors cannot be taken to have a single objective meaning.

## Conclusion

In conclusion, both physical and psychosocial workplace factors are strongly associated with neck and upper limb pain. Potentially, modification or prevention of such risks could appreciably reduce the population burden of neck and upper limb pain. Although exemplified here by the pottery industry in North Staffordshire, these factors are not specific to this industry, and are of likely concern for any occupation involving these physical and psychosocial factors and for any population among which such occupations provide significant levels of employment.

## Competing interests

The author(s) declare that they have no competing interests.

## Authors' contributions

JS was principal investigator, participated in the protocol development, study design, conduct of the study, analysis of the data, writing up and revision of drafts of the manuscript. RL participated in the study design, co-ordination and conduct of the study, data collection, analysis of the data, writing up and revision of drafts of the manuscript. ML participated in the analysis of data, the writing up and revision of drafts of the manuscript. All authors read and approved the final manuscript.

## Pre-publication history

The pre-publication history for this paper can be accessed here:



## Figures and Tables

**Table 1:** Association of neck and upper limb pain with physical activities related to the responders' main job.

	Neck and upper limb pain		
	No	Yes	OR (95% CI)
Repeated lifting of heavy objects			
No*	1229 (56.4%)	950 (43.6%)	1.0
Yes	750 (41.6%)	1054 (58.4%)	1.8 (1.6, 2.1)
Prolonged gripping of object			
No*	1229 (56.8%)	934 (43.2%)	1.0
Yes	750 (41.2%)	1070 (58.8%)	1.9 (1.7, 2.1)
Prolonged bending of neck			
No*	1224 (61.4%)	770 (38.6%)	1.0
Yes	755 (38.0%)	1234 (62.0%)	2.6 (2.3, 3.0)
Repeated finger movements			
No*	871 (55.8%)	690 (44.2%)	1.0
Yes	1108 (45.7%)	1314 (54.3%)	1.5 (1.3, 1.7)
Repeated wrist movements			
No*	963 (58.0%)	697 (42.0%)	1.0
Yes	1016 (43.7%)	1307 (56.3%)	1.8 (1.6, 2.0)
Arms at/above shoulder height			
No*	1483 (54.7%)	1226 (45.3%)	1.0
Yes	496 (38.9%)	778 (61.1%)	1.9 (1.7, 2.2)

*Reference category for calculation of odds ratio; *p *< 0.001 for all risk factors.

**Table 2:** Association of neck and upper limb pain with psychosocial factors related to the responders' main job.

	Neck and upper limb pain		
	No	Yes	OR (95% CI)
Control the way you work			
None of the time*	103 (32.5%)	214 (67.5%)	1.0
A little of the time	117 (37.6%)	194 (62.4%)	0.8 (0.6, 1.1)
Some of the time	331 (46.0%)	388 (54.0%)	0.6 (0.4, 0.7)^#^
Most of the time	653 (53.6%)	566 (46.4%)	0.4 (0.3, 0.5)^#^
All of the time	522 (58.7%)	367 (41.3%)	0.3 (0.3, 0.4)^#^
			
Physically demanding work			
None of the time*	333 (58.5%)	236 (41.5%)	1.0
A little of the time	312 (57.1%)	234 (42.9%)	1.1 (0.8, 1.3)
Some of the time	444 (53.8%)	381 (46.2%)	1.2 (1.0, 1.5)
Most of the time	316 (45.3%)	382 (54.7%)	1.7 (1.4, 2.1)^#^
All of the time	328 (39.3%)	506 (60.7%)	2.2 (1.8, 2.7)^#^
			
Tasks change during time in this job			
None of the time*	441 (48.4%)	470 (51.6%)	1.0
A little of the time	310 (52.3%)	283 (47.7%)	0.9 (0.7, 1.1)
Some of the time	620 (50.7%)	602 (49.3%)	0.9 (0.8, 1.1)
Most of the time	193 (50.5%)	189 (49.5%)	0.9 (0.7, 1.2)
All of the time	157 (45.5%)	188 (54.5%)	1.1 (0.9, 1.4)
			
Job satisfaction			
None of the time*	56 (40.6%)	82 (59.4%)	1.0
A little of the time	105 (47.5%)	116 (52.5%)	0.8 (0.5, 1.2)
Some of the time	297 (48.1%)	321 (51.9%)	0.7 (0.5, 1.1)
Most of the time	835 (53.4%)	729 (46.6%)	0.6 (0.4, 0.8)^#^
All of the time	446 (47.5%)	492 (52.5%)	0.8 (0.5, 1.1)
			
Supportive supervisors/managers			
None of the time*	56 (34.4%)	107 (65.6%)	1.0
A little of the time	150 (43.5%)	195 (56.5%)	0.7 (0.5, 1.0)
Some of the time	346 (46.8%)	394 (53.2%)	0.6 (0.4, 0.8)^#^
Most of the time	708 (52.3%)	645 (47.7%)	0.5 (0.3, 0.7)^#^
All of the time	416 (54.4%)	349 (45.6%)	0.4 (0.3, 0.6)^#^

* Reference category for calculation of odds ratio.

^# ^*p *< 0.05.

**Table 3:** Multivariable analysis of factors associated with neck and upper limb pain.

	Odds ratio (95% CI)*	
	
	Partial model^#^	Full model^#^
Age category		
45–54	2.0 (1.6, 2.3)^§^	2.2 (1.7, 2.7)^§^
55–64	2.4 (2.0, 2.8)^§^	2.7 (2.1, 3.4)^§^
65–75	2.0 (1.7, 2.4)^§^	2.5 (2.0, 3.2)^§^
		
Sex	1.2 (1.1, 1.4)^§^	1.3 (1.1, 1.5)^§^
		
Townsend category		
Moderately affluent	0.9 (0.8, 1.1)	0.9 (0.7, 1.0)
Moderately deprived	1.1 (0.9, 1.3)	0.8 (0.7, 1.0)
Deprived	1.4 (1.1, 1.7)^§^	0.9 (0.7, 1.3)
		
Work activities		
Repeated lifting of heavy objects	2.0 (1.8, 2.3)^§^	1.4 (1.2, 1.7)^§^
Prolonged gripping of object	2.0 (1.8, 2.3)^§^	1.2 (1.0, 1.4)
Prolonged bending of neck	2.6 (2.3, 3.0)^§^	2.0 (1.7, 2.3)^§^
Repeated finger movements	1.5 (1.3, 1.7)^§^	1.0 (0.8, 1.2)
Repeated wrist movements	1.8 (1.6, 2.1)^§^	1.1 (0.9, 1.3)
Arms at/above shoulder height	2.1 (1.8, 2.4)^§^	1.3 (1.1, 1.6)^§^
		
Psychosocial factors^†^		
Little job control	1.9 (1.6, 2.2)^§^	1.6 (1.3, 1.8)^§^
Physically demanding work	1.8 (1.6, 2.1)^§^	1.1 (0.9, 1.3)
Many changes in tasks	1.1 (0.9, 1.3)	1.0 (0.8, 1.2)
Little job satisfaction	1.3 (1.1, 1.5)^§^	0.9 (0.8, 1.1)
Little supervisor support	1.5 (1.3, 1.8)^§^	1.3 (1.1, 1.5)^§^
		
Pottery work as main job	1.3 (1.1, 1.6)^§^	0.9 (0.7, 1.2)

* Odds ratios calculated through i) partial multivariable analysis using logistic regression adjusting for age category, sex, deprivation (Townsend score category), ii) full multivariable analysis using logistic regression adjusting for age category, sex, deprivation (Townsend score category), work tasks, psychosocial factors and pottery work as main job. Reference categories for both models were: '18–44 years' for age; 'Male' for sex; 'Affluent' for Townsend index of deprivation.

^# ^Number of cases analyzed. 1) Partial models: 4994 for age, gender and Townsend categories; 3956 for work activities; between 3342 and 3455 for psychosocial factors; 3956 for pottery work. 2) Full model: numbers analyzed was 3276.

^† ^Psychosocial factors were dichotomized as 'none of the time' or 'a little of the time' or 'some of the time' (reference category for 'physically demanding work' and 'tasks change during time in this job') versus 'most of the time' or 'all of the time' (reference category for 'control the way you work', job satisfaction' and 'supportive supervisors/managers').

^§ ^*p *< 0.05.

**Table 4:** Multivariable analysis of association of work activities and psychosocial factors with individual areas of neck and upper limb pain: neck; shoulder(s); elbow(s); forearm(s), and hand(s).

	Odds ratio (95% CI)*^#^				
	
	Neck	Shoulder(s)	Elbow(s)	Forearm(s)	Hand(s)
Work activities					
Repeated lifting of heavy objects	1.3 (1.0, 1.6)^§^	1.4 (1.1, 1.7)^§^	1.4 (1.1, 1.8)^§^	1.6 (1.2, 2.0)^§^	1.8 (1.4, 2.2)^§^
Prolonged gripping of object	1.1 (0.9, 1.3)	1.0 (0.9, 1.3)	1.2 (1.0, 1.6)	1.2 (0.9, 1.5)	1.6 (1.2, 2.0)^§^
Prolonged bending of neck	2.8 (2.3, 3.4)^§^	2.1 (1.8, 2.5)^§^	1.5 (1.2, 1.9)^§^	1.4 (1.1, 1.8)^§^	1.2 (1.0, 1.5)
Repeated finger movements	0.8 (0.6, 1.0)	1.0 (0.8, 1.2)	1.1 (0.8, 1.4)	1.1 (0.8, 1.4)	1.1 (0.9, 1.5)
Repeated wrist movements	1.0 (0.8, 1.2)	1.0 (0.8, 1.2)	1.2 (0.9, 1.6)	1.4 (1.0, 1.8)^§^	1.1 (0.8, 1.4)
Arms at/above shoulder height	1.2 (1.0, 1.5)	1.1 (0.9, 1.4)	1.0 (0.8, 1.3)	1.1 (0.9, 1.4)	1.1 (0.9, 1.4)
					
Psychosocial factors^†^					
Little job control	1.5 (1.2, 1.8)^§^	1.4 (1.2, 1.7)^§^	1.3 (1.1, 1.7)^§^	1.5 (1.2, 1.8)^§^	1.5 (1.2, 1.8)^§^
Physically demanding work	1.2 (1.0, 1.5)	1.1 (1.0, 1.4)	1.4 (1.1, 1.8)^§^	1.3 (1.0, 1.6)^§^	1.1 (0.9, 1.4)
Many changes in tasks	1.0 (0.8, 1.3)	1.0 (0.8, 1.2)	1.1 (0.8, 1.4)	0.9 (0.7, 1.2)	1.0 (0.8, 1.2)
Little job satisfaction	0.9 (0.8, 1.1)	0.9 (0.7, 1.1)	1.1 (0.8, 1.4)	0.9 (0.7, 1.2)	0.8 (0.6, 1.0)^§^
Little supervisor support	1.3 (1.0, 1.6)^§^	1.2 (1.0, 1.4)	1.1 (0.9, 1.4)	1.1 (0.9, 1.4)	1.3 (1.1, 1.6)^§^

* Odds ratios calculated through logistic regression adjusting for age category, sex, deprivation (Townsend score category), work tasks, psychosocial factors and pottery work as main job.

^# ^Number of cases analyzed was 3276.

^† ^Psychosocial factors were dichotomized as 'none of the time' or 'a little of the time' or 'some of the time' (reference category for 'physically demanding work' and 'tasks change during time in this job') versus 'most of the time' or 'all of the time' (reference category for 'control the way you work', job satisfaction' and 'supportive supervisors/managers').

^§ ^*p *< 0.05.

**Table 5:** Attributable fraction estimates for the association of neck and upper limb pain with work activities and psychosocial factors in the workplace.

	Neck and upper limb pain			
	
	Pain versus no pain		Severe pain versus non-severe pain*	
	AF_ e _^#^	AF_p _^#^	AF_e _^#^	AF_p _^#^
Work activities				
Repeated lifting of heavy objects	15.3 (6.5, 23.1)	7.3 (3.1, 11.1)	-	-
Prolonged bending of neck	27.5 (20.6, 33.8)	13.8 (10.3, 16.9)	23.1 (6.5, 37.1)	11.6 (3.3, 18.6)
Arms at/above shoulder height	9.9 (1.0, 18.7)	3.2 (0.3, 6.0)	16.7 (0.0, 31.0)	5.4 (0.0, 10.0)
				
Psychosocial factors				
Little job control	16.0 (8.3, 22.5)	6.7 (3.5, 9.4)	21.3 (7.4, 33.3)	8.9 (3.1, 13.9)
Physically demanding work	-	-	40.5 (26.5, 51.9)	17.3 (11.3, 22.1)
Little supervisor support	12.3 (3.8, 20.0)	5.1 (1.6, 8.4)	-	-

* Non-severe pain = no pain, or mild pain, or moderate pain.

^# ^Estimates are given as percentages (95% confidence intervals), adjusted for spare-time activities, previous neck/upper limb injury, duration of main job, time since end of main job, and coexisting knee, hip or low back pain. Only statistically significant estimates are shown. See text for definitions of AF_p _and AF_e_.
